# Seamless analysis of liquid samples by coupling a thermal desorption chip with ion mobility spectrometry

**DOI:** 10.1007/s00216-025-06023-7

**Published:** 2025-07-28

**Authors:** Annika Fechner, Carolin Drees, Wolfgang Vautz, Stefanie Sielemann, Ursula Telgheder, Joachim Franzke, Sebastian Brandt

**Affiliations:** 1 Leibniz-Institut für Analytische Wissenschaften – ISAS – e.V., Bunsen-Kirchhoff-Straße 11, 44139 Dortmund, Germany; 2 ION-GAS GmbH, Konrad-Adenauer-Allee 11, 44139 Dortmund, Germany; 3 Hochschule Hamm – Lippstadt – HSHL, Marker Allee 76-78, 59063 Hamm, Germany; 4 Universität Duisburg-Essen – UDE, Forsthausweg 2, 47057 Duisburg, Germany

**Keywords:** Ion mobility spectrometry, Liquid samples, Preconcentration, Thermal desorption, Pre-separation, Trace analysis

## Abstract

A novel coupling of a miniaturized thermal desorption chip (TDC) with a flexible microtube plasma (FµTP) ionization source and ion mobility spectrometry (IMS) enables direct, solvent-free analysis of a liquid sample. The integrated TDC-FµTP-IMS system combines sample introduction, preconcentration, and time-resolved desorption via adjustable temperature gradients. Quantitative analysis of a ketone mixture demonstrated excellent linearity and detection limits between 34 µg L^−1^ and 79 µg L^−1^. In addition, *N*-hexanoyl homoserine lactone was successfully quantified from a liquid sample without manual preparation. The method provides a fast, sensitive, and selective approach for direct liquid-phase IMS applications.

**Graphical abstract:**

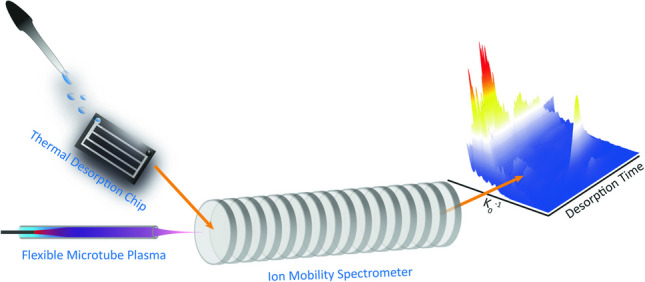

## Introduction

Ion mobility spectrometry (IMS) is a rapid and sensitive technique for the detection of volatile organic compounds (VOCs) and trace gases [1]. It separates gaseous analytes based on their drift velocities in an electric field, which depends on size, shape, and collision cross-section. Since its introduction in the 1970 s [2], IMS has found applications in the detection of chemical warfare agents [3] and explosives [4], and—when coupled with gas chromatography—in more complex tasks such as food quality control [5], forensic analysis [6], and medical diagnostics [7]. Its speed, portability, and low operating cost make IMS highly attractive for field use [8].

However, one major limitation remains: IMS is generally restricted to gaseous analytes. The analysis of liquid samples typically requires intermediate steps such as headspace (HS) sampling [9–11], solid-phase microextraction (SPME) [12] or ionization techniques like Electrospray Ionization (ESI) [13] and Paperspray Ionization (PSI) [14]. These approaches are often complex, limited in compound range, or not suited for portable use. For example, HS is restricted to volatile compounds, SPME and ESI need solvents and complex setups, while PSI depends on disposable carriers and works best with polar substances [15].

As a result, there is a clear need for a compact, solvent-free IMS system capable of directly analyzing liquid samples—especially for on-site applications such as food safety screening, process monitoring, medical point-of-care testing, and environmental fieldwork. Recent developments in miniaturized chip-based systems offer promising solutions. Chip-based high-performance liquid chromatography-IMS [16] and electrochromatography-IMS have shown potential for liquid sample analysis.

A proof-of-concept system is presented that enables the direct analysis of liquid samples by coupling a thermal desorption chip (TDC) with ion mobility spectrometry (IMS) and a non-radioactive flexible microtube plasma (FµTP) ionization source. Through controlled heating, thermal desorption transfers analytes from an adsorbent material into the gas phase, eliminating the need for solvent-based sample introduction. The miniaturized TDC, originally introduced by Zampolli et al. in 2009 [17], integrates enrichment and desorption in a single device with precise temperature control [18]. This dual functionality allows not only direct sample introduction but also preconcentration and time-resolved desorption of analytes—a unique feature that distinguishes the TDC from other liquid sample introduction methods. Previous applications of TDC-IMS combinations have been limited to gaseous samples [19].

The approach described here extends the concept to direct liquid-phase analysis and overcomes key limitations of existing methods, such as solvent dependency, reliance on disposable materials, and restriction to highly volatile compounds. The resulting TDC-IMS platform is compact, solvent-free, compatible with both polar and nonpolar analytes, and utilizes a nonradioactive flexible microtube plasma (FµTP) ionization source, eliminating the need for radioactive materials such as ^63^Ni or ^3^H.

The results demonstrate the feasibility of direct liquid-phase IMS analysis using selected model compounds and form a basis for future developments toward portable analytical systems.

## Materials and methods

### Chemicals and sample preparation

Methanol (purity ≥ 99.8%; HiPerSolv Chromanorm®, VWR Chemicals, Radnor, USA) was used as the solvent for all sample preparations unless otherwise specified. The ketone mixture contained 2-heptanone and 2-octanone (purity 99% and 97%, respectively; Sigma-Aldrich, St. Louis, USA), as well as 2-nonanone and 2-decanone (purity 99% and 98%, respectively; Fluka, Buchs, Austria). The concentrations and boiling points of the individual ketones are summarized in Table 1.

N-Hexanoyl homoserine lactone (Cayman Chemical Company, Ann Arbor, USA) was used as a representative biological compound. All samples were prepared and applied in liquid form.

Unless stated otherwise, all experiments were performed in triplicate.

**Table 1:** Boiling point and concentration of ketone mixture

Compound	Boiling point (°C)	Concentration (mg mL^−1^)
2-Heptanone	151	0.29
2-Octanone	173	0.45
2-Nonanone	195	0.55
2-Decanone	211	0.67

### Thermal desorption chip (TDC)

The thermal desorption chip (TDC) was custom fabricated by the CNR Institute for Microelectronics and Microsystems (Bologna, Italy). The chip consists of a microstructured silicon substrate sealed with a borosilicate glass lid, which integrates both the sample and carrier gas inlets and outlets. Internally, the chip contains microcavities (1 × 1 mm) filled with 36 mg of adsorbent, with the outlet covered by a fine mesh structure to retain the packing material.

The TDC incorporates an integrated heating element and a temperature sensor based on thin-film platinum metallization. The chip is mounted on a miniaturized control unit that includes temperature regulation and active cooling via integrated fans, enabling reduced cycle times. Further technical details on the TDC setup are described by Liedtke et al. [19].

The TDC inlet and outlet are connected to fused silica capillaries (inner diameter: 250 µm; outer diameter: 360 µm). A continuous nitrogen stream (purity: 99.9999%; Linde GmbH, Pullach, Germany) at a flow rate of 200 mL min^−1^ is used as the carrier gas to flush the internal channels of the TDC.

### Adsorbent

Adsorbent materials are generally classified into three categories: carbon-based adsorbents (type I), inorganic adsorbents (type II), and polymer-based adsorbents (type III) [24]. In this study, the type III adsorbent Tenax® TA [poly(2,6-diphenyl-p-phenylene oxide)] (mesh size 80/100; Supelco, Bellefonte, USA) was selected due to its high thermal stability (up to 400 °C), hydrophobic character, and strong adsorption affinity and capacity for non-polar aliphatic and aromatic hydrocarbons. In contrast, it shows low affinity toward water and low-boiling alcohols [20]. The adsorption behavior of Tenax® for gaseous analytes follows the Langmuir isotherm model, which has been extensively studied and described in the literature [21–23].

### Flexible microtube plasma

The flexible microtube plasma (FµTP) ionization source consists of a T-piece assembly, in which a tungsten wire electrode (outer diameter: 75 µm) and the plasma gas supply are connected to a flexible fused silica capillary (inner diameter: 250 µm; outer diameter: 360 µm). The structural design and operational principles of the FµTP have already been described in detail [24]. Helium (purity: 99.999%; Linde GmbH, Pullach, Germany) was used as the plasma gas, supplied at a constant flow rate of 10 mL min^−1^ via a mass flow controller (MFC 0–10 sccm, Analyt-MTC, Müllheim, Germany). Plasma excitation was achieved using a custom-built square-wave high-voltage generator (ISAS e.V., Dortmund, Germany), which produced a rectangular waveform with an amplitude of 1.8 kV, a base frequency of 20 kHz, and a duty cycle of 95:5 (high:low).

### Ion mobility spectrometer

**Fig. 1:**
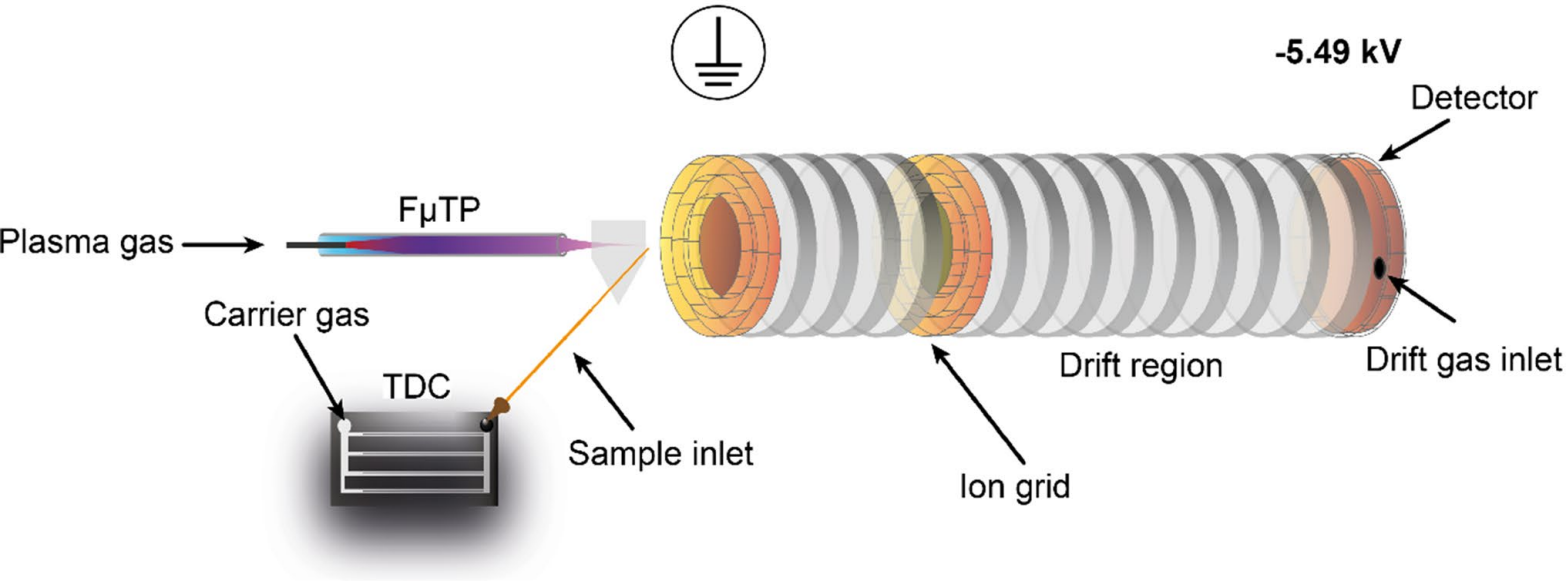
Scheme of the TDC-FµTP-IMS. The structure consists of the TDC, aligned to the FµTP by help of a 3D-printed sample chamber. The chamber is connected to the desolvation region (consisting of a grounded grid, the potential rings and the desolvation gas inlet) and subsequently to the IMS (consisting of the ion gate, the drift region with potential rings, the detector with high-voltage source, and the drift gas inlet)

A custom-built ion mobility spectrometer developed by ISAS e.V. (Dortmund, Germany) was used as the detector (see Fig. 1).

The instrument comprises a drift region (length: 120 mm; inner diameter: 15.2 mm) and an additional desolvation region (length: 80 mm).

Synthetic air (purity: 99.999%; Linde GmbH, Pullach, Germany) was supplied as both drift gas (150 mL min^−1^) and desolvation gas (100 mL min^−1^). At the entrance of the desolvation region, a grounded grid (wire diameter: 0.1 mm; mesh size: 0.3 mm) serves as a counter electrode for the FµTP ionization source. Ion injection is controlled by a Bradbury-Nielsen ion shutter (opening time: 300 µs), and ions are detected by a Faraday plate at the end of the drift region. The IMS operates with a cycle time of 50 ms.

The high-voltage power supply is connected to the detector and set to − 5.49 kV DC, allowing the detection of positive ions. A homogeneous electric field is applied across both the drift and desolvation regions, with a field strength of 27.4 V mm^−1^. All measurements were conducted at ambient temperature and pressure.

### Liquid sampling

**Fig. 2:**
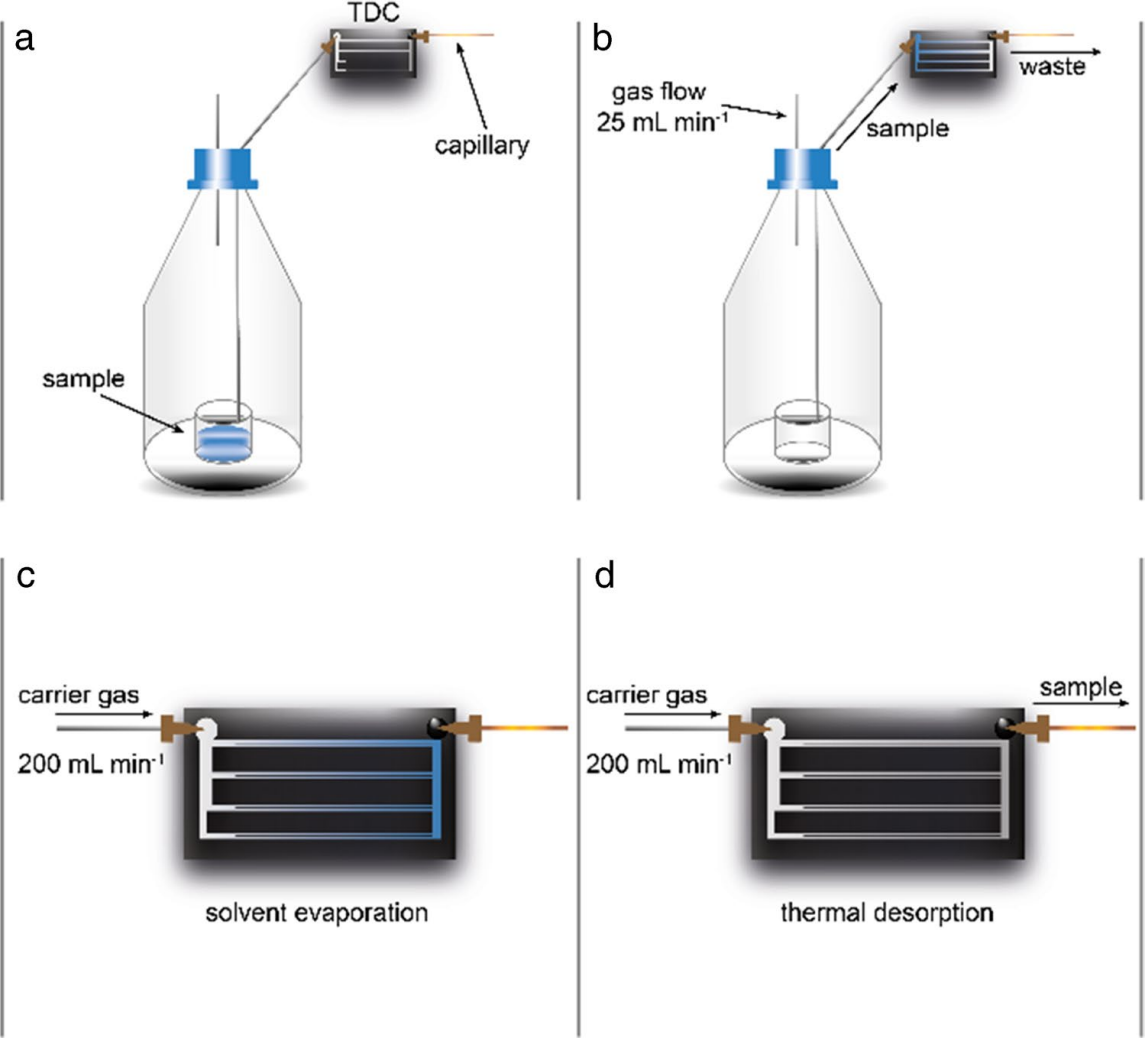
Scheme of the TDC. **Loading**: A sample vial is connected to the TDC (**a**) and a sample gas flow is applied (**b**) for flushing the liquid sample through the TDC adsorbent and the non-adsorbed sample components into a waste container. **Evaporation**: The TDC is heated for a specified time and carrier gas flow (**c**). **Desorption**: The TDC is connected to the IMS sample inlet. During a controlled temperature program, adsorbed molecules are desorbed and transported to the FµTP-IMS (**d**)

The liquid sample introduction procedure is illustrated in the flow chart shown in Fig. 2. A sample volume of 0.5 mL is transferred into a 25 mL headspace vial (Schott, Germany), which is sealed gas-tight and connected to the TDC inlet (Fig. 2a). A nitrogen carrier gas (flow rate: 25 mL min^−1^) transports the vaporized analytes into the internal channels of the TDC (Fig. 2b). During the loading phase, the TDC is maintained at a constant operating temperature of 30 °C. Non-adsorbed components are purged through the outlet and directed to waste.

Subsequently, the TDC is flushed with nitrogen at an increased flow rate of 200 mL min^−1^ for a defined period to remove residual matrix components (Fig. 2c). For analysis, the outlet of the TDC is switched from waste to the IMS inlet, and thermal desorption is initiated by applying a programmed temperature gradient (Fig. 2d). The specific temperature profile is selected based on the boiling points of the target analytes to ensure optimal desorption efficiency.

### Headspace method

Headspace measurements were conducted by heating the sample vial to 80 °C. Sample introduction into the IMS system was achieved using a directional valve and a 9 mL sample loop. Deionized water (VeH_2_O) served as the solvent.

### Data acquisition, evaluation and visualization software

Data acquisition was performed using the software *qIMS* (ISAS e.V., Dortmund, Germany). Data processing, including the calculation of reduced ion mobilities, evaluation, and visualization, was conducted using *IONysos* (ION GAS GmbH, Dortmund, Germany). All graphical illustrations and calibration curve fittings were generated with *Origin 202* (OriginLab Corporation, Northampton, USA).

### Determination of the limit of detection (LOD) using the Hubaux-Vos method

The limit of detection (LOD) was determined using the Hubaux-Vos method, which offers a statistically robust approach based on linear calibration [25]. This method involves constructing a calibration curve with a 95% confidence interval. The signal threshold is defined by the intersection of the upper confidence limit with the y-axis. The corresponding LOD is then obtained by projecting from this point along the lower confidence boundary until it intersects the regression line. The x-coordinate of this intersection represents the detection limit for the respective analyte.

## Results and discussion

A combined TDC-FµTP-IMS system was developed and optimized in this study. During the initial coupling, signal instabilities were observed, caused by interferences between the square-wave high-voltage of the FµTP and the drift voltage with pulsed ion gate of the IMS, which negatively affected the measurement process.

To mitigate these interferences, the IMS system was adapted as described in the “Ion mobility spectrometer” section. Specifically, the polarity and position of the IMS drift voltage supply were modified by relocating the high-voltage input from the IMS inlet side to the detector side, effectively decoupling it from the FµTP. In addition, a short desolvation region was introduced between the ion gate and the ionization zone to minimize mutual influence. A grounded grid was placed at the entrance of this desolvation region, serving as a counter electrode for the FµTP and further stabilizing the ionization process.

Following the successful integration of the TDC-FµTP-IMS system, further optimization was carried out. The focus was placed on achieving short sampling times, low carrier gas flow rates, high sensitivity, and efficient evaporation. For these optimization steps, a homologous series of ketones—2-heptanone, 2-octanone, 2-nonanone, and 2-decanone—was used as a model analyte mixture.

### TDC evaporation time

Evaporation is initiated immediately after introducing the sample into the TDC. For this purpose, carrier gas is flushed through the internal channels of the TDC, while the chip is heated to a temperature slightly above the boiling point of the solvent used (see the “TDC carrier gas flow” section). The evaporation period is designed to ensure complete removal of residual solvent while minimizing analyte loss.

**Fig. 3:**
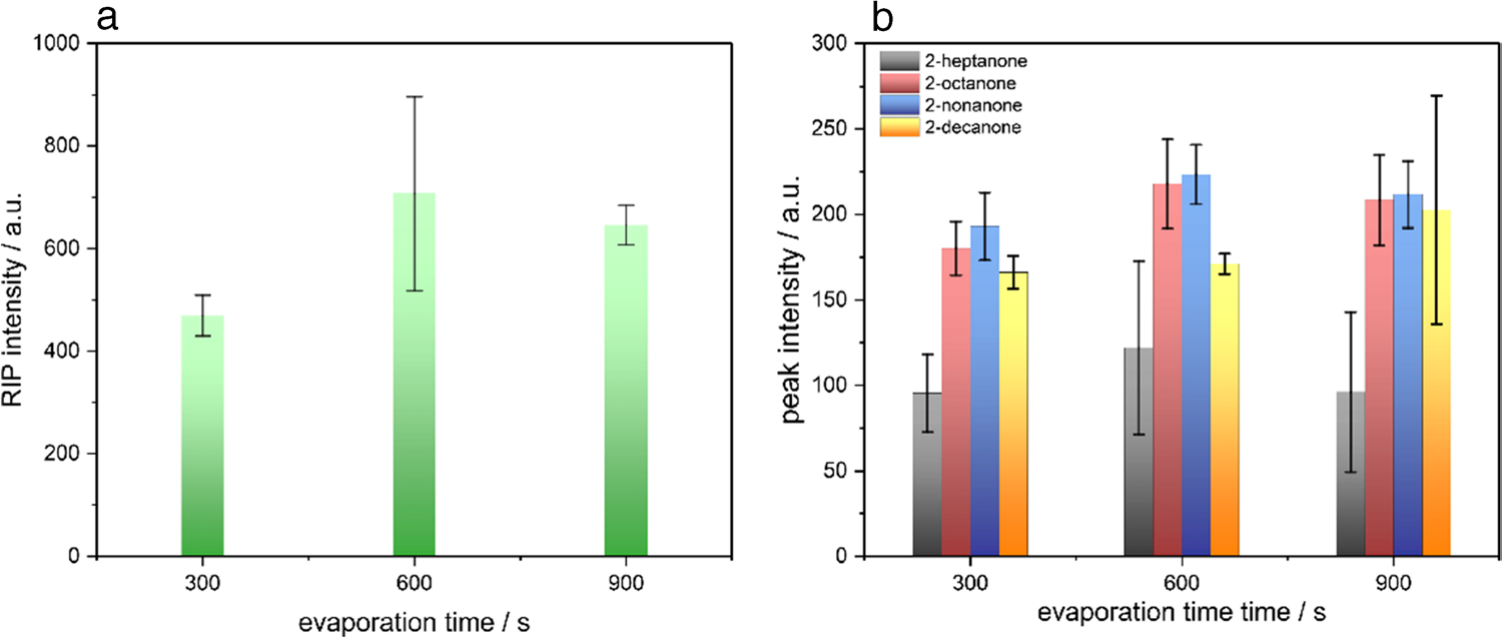
Intensity of the RIP in dependence of the TDC evaporation time (**a**). Monomer signal intensities for four ketones (2-heptanone, 2-octanone, 2 nonanone and 2-decanone) in dependence of the evaporation time (**b**)

To monitor and optimize the evaporation process prior to thermal desorption, both the signal intensity of the reactant ion peak (RIP; Fig. 3a) and the analyte-specific ketone signals (Fig. 3b) were evaluated as performance indicators. Three evaporation durations (300 s, 600 s, 900 s) were systematically compared.

An evaporation time of 600 s yielded the highest RIP intensity, suggesting a more complete removal of residual solvent and thereby reduced chemical background. However, this increase in RIP did not correlate with higher analyte signals, as the peak intensities of the individual ketones remained largely consistent across all three conditions. This observation indicates that prolonging the evaporation beyond 300 s did not lead to enhanced desorption efficiency or improved ionization of the analytes. Notably, the shortest evaporation time of 300 s resulted in the lowest standard deviations for the analyte peaks, indicating more stable and reproducible measurements. This is likely due to reduced sample loss from prolonged heating or evaporation variability. In balancing signal stability, analysis time, and practical throughput, 300 s was therefore selected as the optimal evaporation duration for all subsequent experiments.

### TDC carrier gas flow

**Fig. 4:**
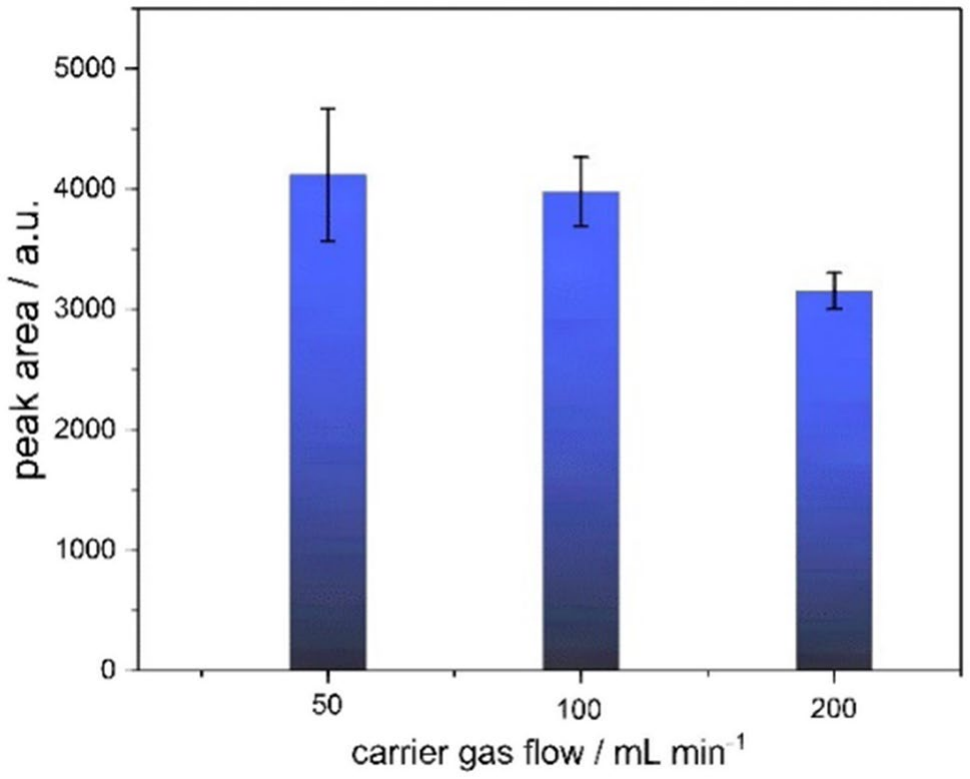
Monomer signal intensity (area) of 2-octanone for TDC carrier gas flow rates of 50, 100, and 200 mL min^−1^ as bar chart

Three different carrier gas flow rates (50–200 mL min^−1^) were evaluated for their influence on analyte signal intensity within the TDC. The effects of varying flow rates were assessed using the monomer signal of 2-octanone as a representative analyte (Fig. 4). A decrease in signal intensity was observed with increasing flow rates, amounting to approximately 20% signal loss at 200 mL min^−1^, likely due to dilution effects in the ionization region. However, standard deviations decreased with higher flow rates, indicating improved measurement precision. Considering both signal intensity and reproducibility, a carrier gas flow rate of 200 mL min^−1^ was selected for subsequent measurements, as it yielded the lowest relative standard deviation (4.7%).

### Comparison of TDC with headspace sampling method

**Fig. 5:**
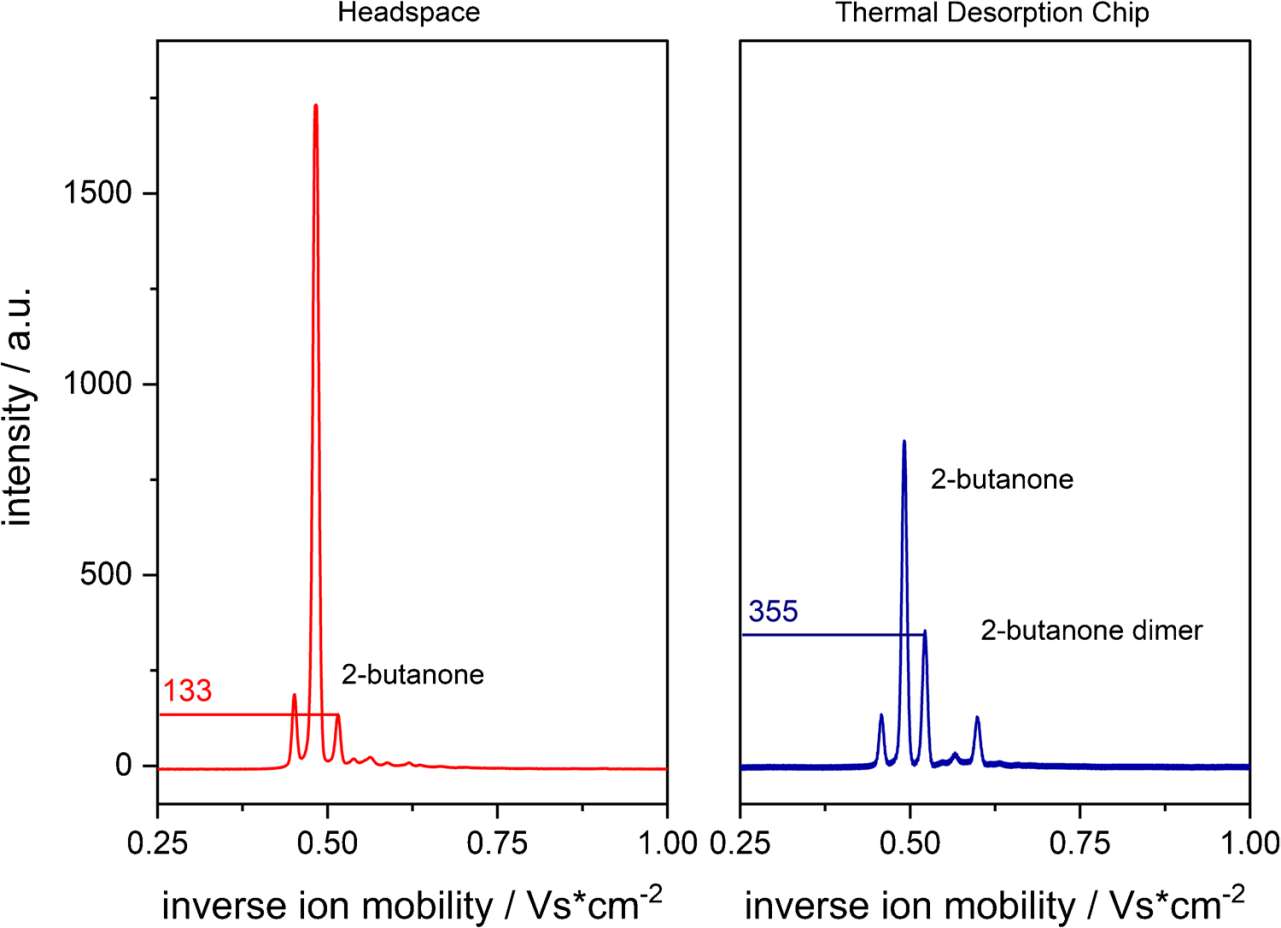
Detection of 2.5 µg L^−1^ 2-butanone using (red) HS-FµTP-IMS and (blue) TDC-FµTP-IMS. Signal intensities are marked in red for HS-FµTP-IMS and in blue for TDC-FµTP-IMS on the ordinate. In addition to the monomer, the TDC-FµTP-IMS measurement also reveals the corresponding dimer signal

To benchmark the performance of TDC-based sample introduction against conventional HS sampling, a 2.5 µg L^−1^ dilution of 2-butanone in deionized water (VeH_2_O) was analyzed using both methods under otherwise identical conditions. The resulting IMS spectra (Fig. 5) reveal clear performance differences between the two approaches.

The TDC-FµTP-IMS setup produced more than twice the signal intensity compared to the HS-FµTP-IMS configuration, demonstrating a markedly higher sensitivity for the detection of low-concentration analytes. This enhanced performance is likely attributable to the combination of analyte preconcentration and controlled thermal desorption provided by the TDC, which facilitates a more efficient transfer of analytes into the gas phase and subsequently into the ionization region.

In addition, the TDC-based method enabled the detection of both monomer and dimer signals of 2-butanone, whereas the HS approach yielded only the monomer signal.

### TDC analyte volume

The adsorptive behavior of Tenax® for liquid-phase samples and its impact on method sensitivity were investigated by varying the sample volume. Aqueous solutions of 2-octanone at a constant 2.5 µg L^−1^ concentration were prepared in purified water and applied in volumes ranging from 0.25 mL to 1.00 mL. Thermal desorption and detection were performed using the TDC-FµTP-IMS setup.

**Fig. 6:**
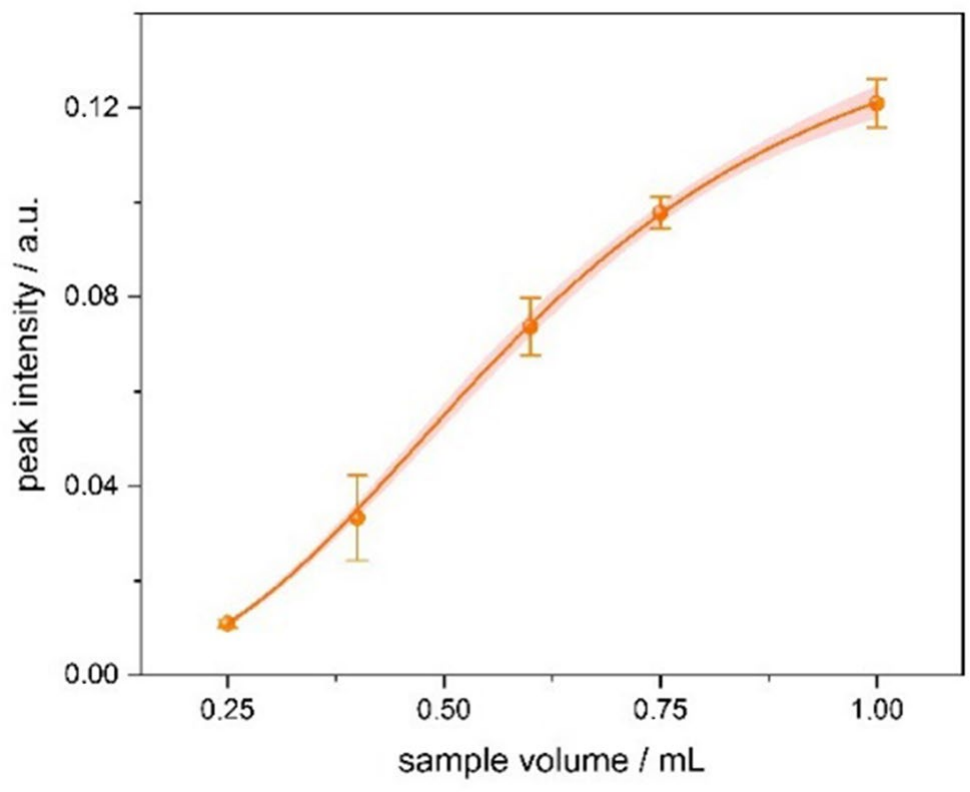
Peak intensity of 2.5 µg L^−1^ 2-octanone in aqueous solution for sample volumes between 0.25 mL and 1.00 mL, illustrating the adsorption capacity of Tenax® in the TDC

Water was chosen as the solvent due to the hydrophobic nature of Tenax®, which facilitates efficient removal of excess water, even at higher volumes. As shown in Fig. 6, increasing the sample volume by a factor of 4 (from 0.25 mL to 1.00 mL) resulted in a 12-fold increase in signal intensity, underlining the strong preconcentration capability of the system. The relationship between sample volume and signal intensity follows the Langmuir isotherm model, with a coefficient of determination (R^2^) of 0.99, indicating the liquid. However, beyond a sample volume of 1, the increase in signal plateaus, suggesting saturation of the adsorbent and the onset of a breakthrough point. This defines a practical upper limit for sample volume in routine applications to avoid signal loss or distortion due to overloading.

### TDC pre-separation

Due to its precise thermal control and miniaturized design, the TDC can be used as a simple pre-separation unit through the application of tailored temperature gradient programs. The combination of heating rate, analyte boiling points, and desorption behavior enables a semi-resolved time-dependent separation.

**Fig. 7:**
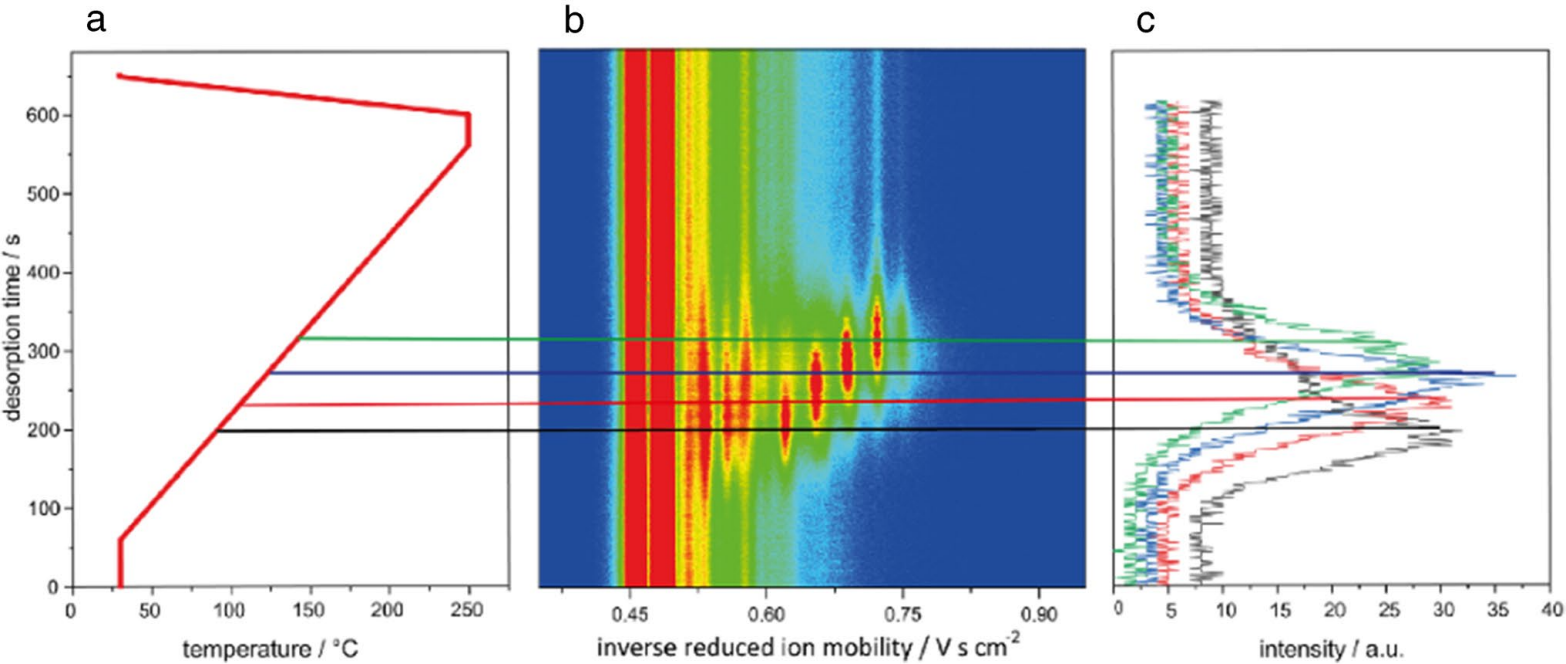
**a** Applied temperature program for the TDC; **b** IMS spectra of the ketone mixture shown as a heatmap with color-coded signal intensities; **c** corresponding extracted ion mobility chromatograms for 2-heptanone (black), 2-octanone (red), 2-nonanone (blue), and 2-decanone (green)

The temperature program applied to the ketone mixture is shown in Fig. 7a. Starting from room temperature, the TDC was heated to 30 °C (held for 60 s), followed by a linear temperature ramp at 0.44 °C s^−1^ up to 250 °C (held for 10 s). A subsequent cooling step (~ 60 s) ensures rapid readiness for the next measurement. Due to the high degree of miniaturization, total measurement time was reduced to approximately 11 min.

Depending on the required resolution and sample characteristics, the temperature program can be adjusted, as the TDC enables programmable heating rates between 0 °C s^−1^ and 50 °C s^−1^ and supports maximum operation temperatures of up to 250 °C. This flexibility allows for application-specific adaptation of desorption times, ranging from rapid screening to selective pre-separation. The thermal desorption behavior of the ketones is illustrated in Fig. 7b and c. Each compound desorbed at a characteristic time point based on its boiling point, allowing effective pre-separation and individual identification. Within 6.67 min, all ketones were thermally resolved and identified by their inverse ion mobilities in comparison to reference values from the ISAS e.V. database (Table 2).

**Table 2:** Measured desorption temperature and K_0_^−1^ of the ketone mixture

Component	K_0_^−1^	Desorption temperature in °C
2-Heptanone	0.62	88
2-Octanone	0.65	102
2-Nonanone	0.69	115
2-Decanone	0.73	125

### Quantitative evaluation of ketones via standard addition

**Fig. 8:**
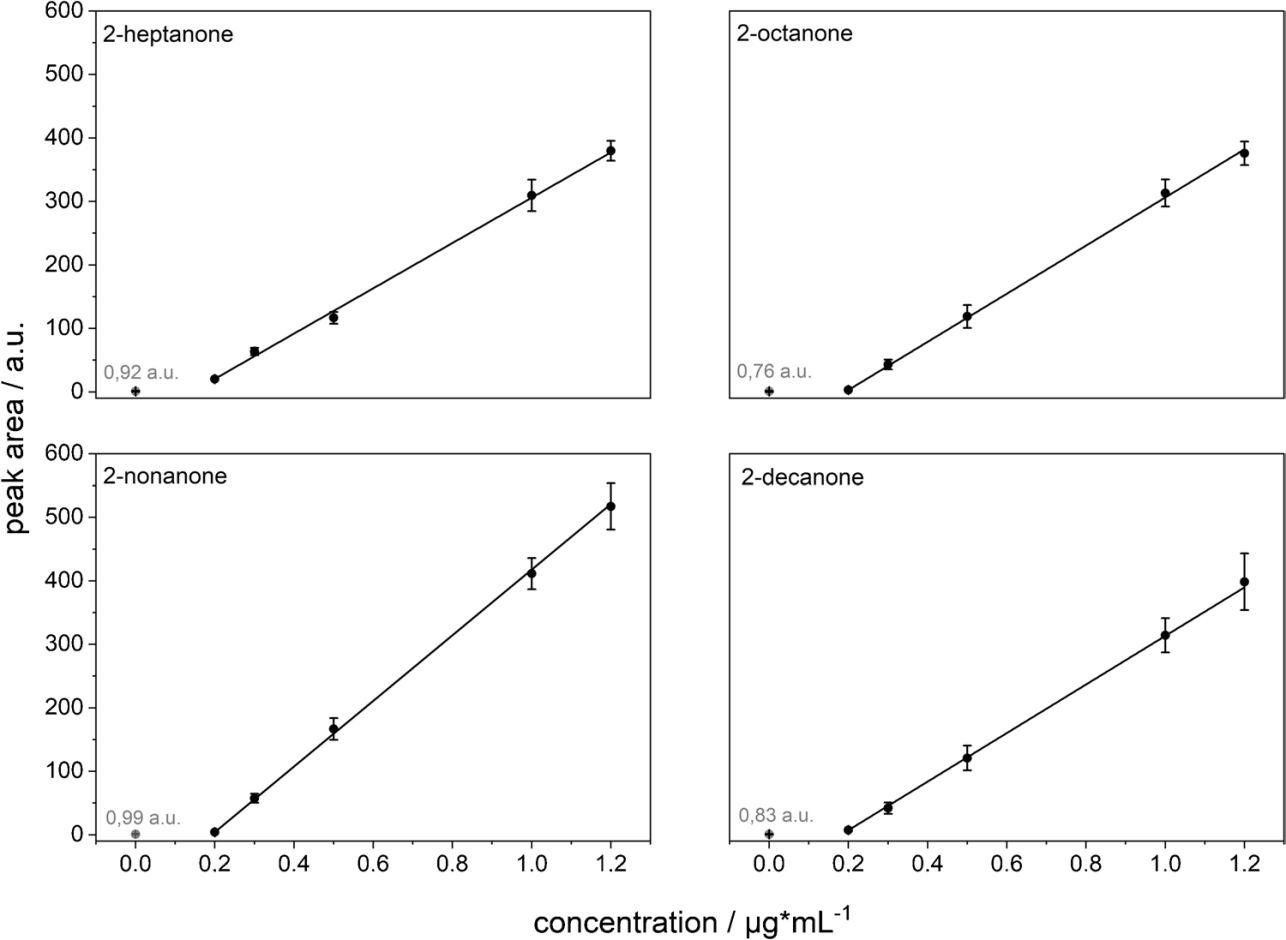
Standard addition calibration curves for the quantification of 2-heptanone, 2-octanone, 2-nonanone, and 2-decanone using the TDC-FµTP-IMS system. Each point represents the mean of triplicate measurements, with error bars indicating the standard deviation. Procedural blanks (0 µg mL^−1^) were included and are displayed as open symbols

Quantitative analysis of the ketone mixture was performed using the TDC-FµTP-IMS system as described in the “TDC pre-separation” section. Calibration was based on the standard addition method to account for potential matrix effects and to ensure accurate quantification directly from the liquid phase. The analytes—2-heptanone, 2-octanone, 2-nonanone, and 2-decanone—were analyzed in triplicate, and the resulting calibration curves are shown in Fig. 8. All compounds exhibited excellent linearity across the tested concentration range (0.2–1.2 mg L^−1^), with coefficients of determination (R^2^) ≥ 0.999.

Procedural blanks (0 µg mL^−1^) were included in each calibration and are displayed as open symbols in Fig. 8. The integration of these blank values allowed for robust baseline correction and visual validation of background signal suppression. Relative standard deviations ranged from 2.8% (at 0.3 mg L^−1^ 2-decanone) to 21.1% (at 1.2 mg L^−1^ 2-heptanone), indicating good repeatability within the tested range.

Limits of detection (LODs) were calculated using the Hubaux-Vos method (see the “Determination of the limit of detection (LOD) using the Hubaux-Vos method” section), which determines the detection limit based on the intersection of confidence intervals with the calibration function. The resulting LODs ranged from 34 µg L^−1^ (2-nonanone) to 79 µg L^−1^ (2-heptanone), as summarized in Table 3. To evaluate the system’s performance across varying compound characteristics, a homologous series of four ketones was chosen. Their differences in volatility and affinity to the sorbent material provide a representative basis for assessing the method’s responsiveness to diverse physicochemical properties.

**Table 3:** Determined limits of detection (LOD) of the compounds of the ketone mixture

Compound	LOD (mg L^−1^)
2-Heptanone	0.78
2-Octanone	0.48
2-Nonanone	0.34
2-Decanone	0.40

In addition to sensitivity, the method’s accuracy was assessed through recovery experiments using the standard addition approach. Recoveries for 2-octanone (98.3%) and 2-nonanone (92.7%) confirmed high analytical accuracy and efficient desorption and ionization. 2-decanone showed a comparable recovery of 93.0%, albeit with slightly reduced precision. In contrast, 2-heptanone exhibited a lower recovery rate of 72.1%, which is attributed to its higher volatility and weaker retention on Tenax® TA. These results highlight the influence of compound-specific physicochemical properties on analytical performance.

### TDC-FµTP-IMS for in liquid solution N-hexanoyl homoserine lactone detection

The potential of the TDC-FµTP-IMS system for direct analysis of liquid-phase samples without additional sample preparation was demonstrated through the detection of *N*-hexanoyl homoserine lactone (C6-HSL) in methanol. For this purpose, a calibration curve series of C6-HSL solutions was prepared and analyzed using thermal desorption.

**Fig. 9:**
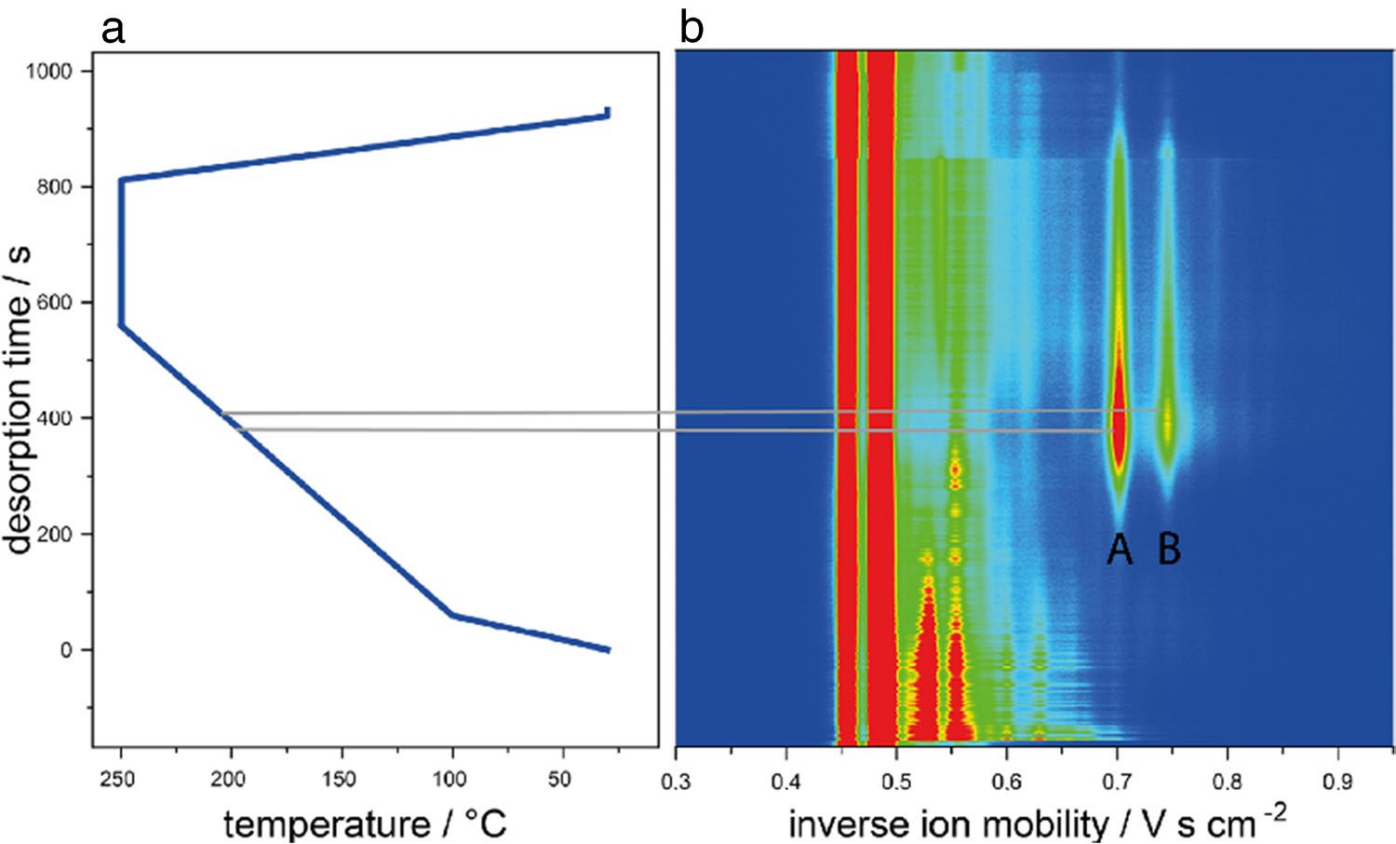
**a** Thermal desorption temperature program optimized for C6-HSL; **b** IMS heatmap of 0.75 mg L^−1^ C6-HSL in methanol. Signal A (K₀^−1^ = 0.70, 208 °C) and signal B (K₀^−1^ = 0.74, 197 °C) are assigned to C6-HSL. Signals between K₀^−1^ = 0.5–0.6 are attributed to background contamination

The standard temperature program used for ketone separation was adapted for C6-HSL to improve desorption efficiency (Fig. 9a). The program began at 30 °C with a heating rate of 1.7 °C s^−1^ up to 100 °C, followed by a slower ramp of 0.3 °C s^−1^ to 250 °C, which was held for 250 s. The TDC was then cooled back to the starting temperature. The total analysis time was approximately 15 min.

The thermal desorption of 0.75 mg L^−1^ C6-HSL in methanol is illustrated in the IMS heatmap (Fig. 9b), showing two distinct signals attributed to C6-HSL: signal A (K₀^−1^ = 0.70, desorption temperature ≈ 208 °C) and signal B (K₀^−1^ = 0.74, desorption temperature ≈ 197 °C). Additional signals observed in the range K₀^−1^ = 0.5–0.6 were assigned to background contaminants.

**Fig. 10:**
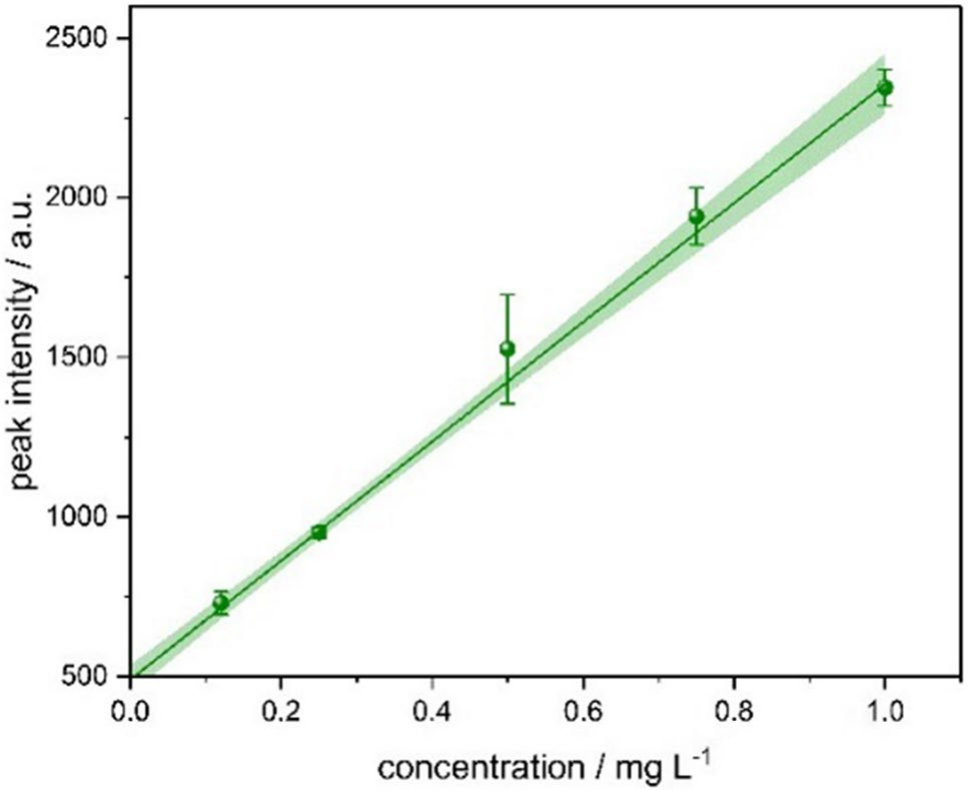
Calibration curve of N-hexanoyl homoserine lactone using TDC-FµTP-IMS. Data represent triplicate means with absolute error bars; the colored line indicates the linear regression, and shaded areas show the 95% confidence interval

For quantitative analysis, signal A was used to establish a calibration curve of C6-HSL. C6-HSL was dissolved in methanol at five different concentrations ranging from 0.12 to 1.00 mg L^−1^. Each concentration was measured in triplicate (Fig. 10). The calibration curve yielded a coefficient of determination of R^2^ = 0.999 with relative standard deviations between 1.8% (at 0.25 mg L^−1^) and 11.2% (at 0.50 mg L^−1^). The calculated limit of detection was 46 µg L^−1^.

## Conclusion and outlook

The TDC-FµTP-IMS system was successfully developed and optimized for the direct analysis of liquid-phase samples. Its modular design and adjustable parameters, such as sample volume and desorption gradient, enable flexible adaptation to various analytical tasks.

Quantitative investigations using a homologous ketone mixture and the biologically relevant analyte C6-HSL confirmed the system’s suitability for direct analysis without additional sample preparation. Detection limits of 34 µg L^−1^ (ketones) and 46 µg L^−1^ (C6-HSL), combined with total analysis times under 15 min, highlight the method’s analytical performance.

While conventional methods like liquid–liquid extraction followed by GC–MS can achieve lower LODs (e.g., 12.9 µg L^−1^ for C6-HSL [26]), they require extensive sample preparation and are prone to handling errors. In contrast, the TDC-FµTP-IMS approach enables fast, solvent-free, and direct analysis. Detection limits could potentially be further improved through increased sample loading or refined desorption conditions.

In summary, the TDC-FµTP-IMS platform offers a compact and versatile alternative to electrospray ionization, paper spray, or headspace-based IMS systems. It unites sample introduction, enrichment, desorption, and separation in a single setup and supports both polar and non-polar analytes.

Although this proof-of-concept study focused on model compounds under controlled conditions, real sample validation remains an important next step. The deliberate use of defined analytes allowed for systematic method development and performance evaluation. Future work will extend the approach to complex matrices to assess matrix effects, selectivity, and robustness under realistic conditions. These efforts will also support the development of automated and mobile systems for on-site diagnostics.

## Data Availability

All data generated or analyzed during this study are included in this published article. Further details are available from the corresponding author on reasonable request.

## Abbreviations

C6-HSL: *N*-Hexanoyl homoserine lactone
ESI: Electrospray ionization
FµTP: Flexible microtube plasma
HV: High voltage
ID: Inner diameter
IMS: Ion mobility spectrometry
LOD: Limit of detection
MS: Mass spectrometry
OD: Outer diameter
PSI: Paperspray ionization
RIP: Reactant ion peak
SPME: Solid-phase micro extraction
TDC: Thermal desorption chip
VOC: Volatile organic compounds
